# Copy Number Variants Contributing to Combined Pituitary Hormone Deficiency

**DOI:** 10.3390/ijms21165757

**Published:** 2020-08-11

**Authors:** Bartłomiej Budny, Katarzyna Karmelita-Katulska, Marek Stajgis, Tomasz Żemojtel, Marek Ruchała, Katarzyna Ziemnicka

**Affiliations:** 1 Department of Endocrinology, Metabolism and Internal Diseases, Poznan University of Medical Sciences, 60-355 Poznan, Poland; mruchala@ump.edu.pl (M.R.); kaziem@ump.edu.pl (K.Z.); 2 Department of General Radiology and Neuroradiology, Poznan University of Medical Sciences, 60-355 Poznan, Poland; kkatulska@ump.edu.pl (K.K.-K.); mstajgis@ump.edu.pl (M.S.); 3 Genomics Platform, Berlin Institute of Health, 10117 Berlin, Germany; tomasz.zemojtel@bihealth.de; 4 Institute of Bioorganic Chemistry, Polish Academy of Sciences, 60-569 Poznan, Poland

**Keywords:** CPHD, CNVs, microarrays

## Abstract

Combined pituitary hormone deficiency represents a disorder with complex etiology. For many patients, causes of the disease remain unexplained, despite usage of advanced genetic testing. Although major and common transcription factors were identified two decades ago, we still struggle with identification of rare inborn factors contributing to pituitary function. In this report, we follow up genomic screening of CPHD patient cohort that were previously tested for changes in a coding sequences of genes with the use of the whole exome. We aimed to find contribution of rare copy number variations (CNVs). As a result, we identified genomic imbalances in 7 regions among 12 CPHD patients. Five out of seven regions showed copy gains whereas two presented losses of genomic fragment. Three regions with detected gains encompassed known CPHD genes namely *LHX4, HESX1*, and *OTX2*. Among new CPHD loci, the most interesting seem to be the region covering *SIX3* gene, that is abundantly expressed in developing brain, and together with *HESX1* contributes to pituitary organogenesis as it was evidenced before in functional studies. In conclusion, with the use of broadened genomic approach we identified copy number imbalances for 12 CPHD patients. Although further functional studies are required in order to estimate its true impact on expression pattern during pituitary organogenesis and CPHD etiology.

## 1. Introduction

The combined pituitary hormone deficiency (CPHD) represents a challenge for clinicians. The complex background of the disease could make diagnostic assignment difficult. The genetic causes for majority of cases are linked to founder mutations in *PROP1* transcription factor gene, and are typical for familial cases [1,2,3]. Mutations in four other transcription factors genes namely *POU1F1, LHX3, LHX4,* and *HESX1* represent a canonical set of defects accounting for significant portion of patients [4,5]. In the era of massive parallel sequencing methods, the number of genes involved in pituitary functioning was broaden dramatically, evidencing a huge complexity of developmental processes [6]. Even though, for numerous cases with clinical features indicative for CPHD, the causative genetic background cannot be identified [7]. Recent research provided new evidence on the cumulative effect of mild variants and oligogenicity, indicating a great abundance of possible contributors affecting pituitary organogenesis, but also evidencing unusual non-mendelian transmission [8,9]. So far, bigger genomic rearrangements bearing multiple genes are responsible for more severe phenotypes, usually syndromic or accompanied with dysmorphisms [10]. Several rearrangements were linked to the pituitary phenotype. The 2p25 duplication with co-existing 2q37 distal deletion was identified in a patient presenting GH deficiency and pituitary abnormalities (hypoplasia, pituitary stalk interruption syndrome (PSIS), posterior lobe ectopy) [11]. In another patient presenting PSIS, the 17q21.31 loss was also found [12]. Significant variability encompassing the *SOX3* gene on Xq26, was detected in familial cases presenting X-linked transmission of hypopituitarism [13,14]. Finally, pathogenic copy number variations of 3q13.31-q13.32, 8q23.1-q24.11, 2p25.3-p24.3, and 4q35.1-q35.2 were found in patients presenting congenital hypopituitarism associated with severe complex phenotypes [15].

In this work, we aim to look for CNVs in milder and less variable phenotype of CPHD. The patients’ cohort was previously screened for pathogenic point mutations (*PROP1* founder mutations and WES study [16]) and we included only patients with so far unexplained causes of the disease.

## 2. Patients

The patient cohort was reported before, in previous publications [16,17]. Briefly, we examined 32 CPHD patients, recruited from the Department of Endocrinology, Metabolism, and Internal Diseases at Poznan University of Medical Sciences. All patients are sporadic, and no familial cases were examined. All showed deficiencies of GH, gonadotropins and TSH. Adrenocorticotrophic hormone deficiency was diagnosed for 14 out of 32 individuals. Prolactin deficiency was diagnosed in four patients. Magnetic resonance imaging (MRI) revealed pituitary malformation in all cases (22 presenting hypoplasia and 10 PSIS). An average age at diagnosis was 10 years (+/−5,3 years) and gender ratio 13F/19M (females/males). We looked for dysmorphic features and abnormalities associated to pathogenic mutations in pituitary genes like craniofacial abnormalities, optic nerve hypoplasia, or vision defects. No such symptoms were diagnosed. The comprehensive information regarding this study as well as genetic examination was provided to all participants and signed informed consent was obtained. In a previous study, we examined this cohort for mutations in pituitary related genes and whole exome. The presented cohort did not reveal any convincingly pathogenic mutations that could be responsible for CPHD phenotype. In order to exclude population specific CNVs, we used a population control cohort with microarray data. All individuals are originated from the same country. The Bioethical Committee of Poznan University of Medical Sciences approved the study (number 441/11, 12 May 2011).

## 3. Methods

All the samples were previously processed and reported showing no pathogenic mutations in CPHD targeted panel of genes and whole exome sequencing (WES). Briefly, genomic DNA was obtained from blood leukocytes using phenol-chloroform method. After extraction, DNA was assessed for concentration and purity using NanoDrop spectrophotometer. Affymetrix Human CytoScanHD and CytoScan750K Array (Thermo Fisher Scientific, Waltham, MA, USA) were used to detect rearrangements that are undetectable using WES. For each sample, a 250 ng of genomic DNA was processed according to the manufacturer’s protocol. First step assumed digestion with the restriction enzyme *Nsp*I, following by ligation to short nucleotide adapters. Adapters were fixed using PCR (separate primers matching the adapter sequence). Amplicons were inspected on a 2% agarose and afterwards purified using Agencourt AMPure magnetic beads (Beckman Coulter, Brea, CA, USA) according to manufacturer protocol. PCR products were than digested using fragmentation reagent (Thermo Fisher Scientific) and checked on Hi-resolution agarose (4%). Final steps include end-labelling using biotin and overnight 18-h hybridization. For washing and staining chips we used GeneChip^®^ Fluidics Station 450 and for scanning GeneChip^®^ Scanner 3000 7G (Thermo Fisher Scientific, Waltham, MA, USA). Copy number changes were calculated after normalization to baseline reference intensities (Affymetrix model NA 33.3) and hidden Markov model (HMM) implemented to Chromosome Analysis Suite v200159 (ChAS, Affymetrix). Gains were classified as log_2_ratio ≥1.3 and deletions as log_2_ratio ≤0.7. Artificial false positive CNVs were filtered out and only fragments confirmed by at least 10 consecutive probes, exceeding 20 Kbp in size were regarded. Gains and losses were analyzed separately.

In order to confirm unbalanced alterations within known CPHD genes we used MLPA assay conducted with the commercially available kit SALSA MLPA P216 (MRC Holland, Amsterdam, Netherlands). This assay is design to detect structural abnormalities within selected pituitary genes: *GH1, POU1F1, PROP1, GHRHR, LHX3, LHX4*, and *HESX1.* A multiplex, quantitative PCR with FAM-labeled primers was performed to generate fragments between 120 and 500 nt, in the presence of nine control fragments for each patient. Products were then separated on capillary electrophoresis and from generated chromatograms peak areas were calculated. Further analysis included two steps of normalizations: intra-sample (relative value of peaks within one patient, regarding reference fragments) and block-normalization (comparison of peak values between all patients and samples from control individuals). In order to ensure proper data management, we employed Coffalyser script (MRC Holland). A cohort of 20 healthy individuals was additionally used as a reference group for MLPA.

### Filtering Genomic Data

For the filtering the CNVs identified using microarray studies we applied following criteria: (1) rearrangement size gathered in routine cytogenetic screening exceeding 300 Kb; (2) CNVs in between 300 Kb and 20 Kb in size (confirmed by a signal from at least 10 independent probes), fragments shorter than 20 Kb and confirmed by less than 10 probes were filtered out; (3) analysis of CNVs encompassing pituitary genes evidenced to cause disease; (4) focused analysis of CNVs covering genes expressed in pituitary. Polymorphic CNVs were excluded using Database of Genomic Variants (DGV, http://projects.tcag.ca/variation). For gene positions we used ChAS (NA 33.3, Affymetrix) as well as genome browsers USCS (http://genome.ucsc.edu) and Ensembl (http://www.ensembl.org). Human reference sequence was genome build 19 (hg19).

For filtering out population specific variants, we used a cohort of 100 controls originating from the same population. Selected CNVs were confirmed using MLPA and quantitative real-time qPCR (ΔΔCT method, Thermo Fisher Scientific). The selection of 353 pituitary specific genes was accomplished with the use of the protein atlas (https://www.proteinatlas.org/).

## 4. Results

**Figure 1:**
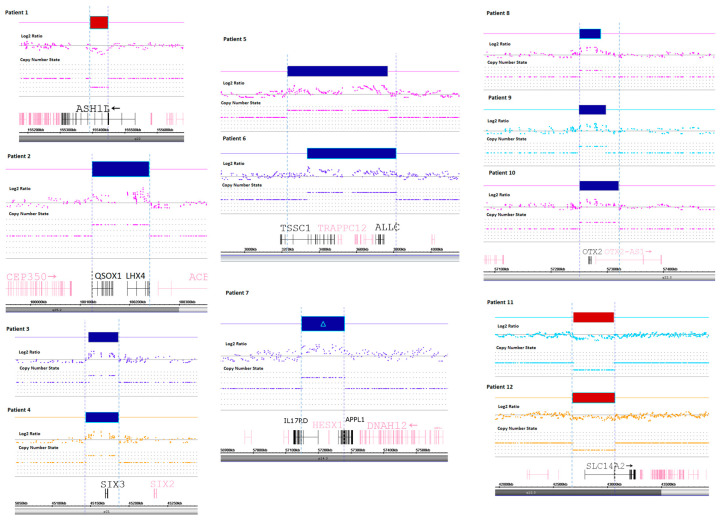
Microarray rearrangements detected in CPHD patients. Detected regions in patients are shown in top section (in red—deletions, in blue—duplications), followed by Log2 Ratio plot, copy number state, gene map and cytoband (bottom section). Patient numbers correspond to those presented in Table 1 and Table 2.

The research strategy assumed identification of genetic abnormalities and imbalances, undetectable using massive sequencing approach. In the first step, we looked for bigger genomic rearrangements (300 Kbp) that were gathered in a routine cytogenetic testing. No such findings were detected. In a second step, we focused on known CPHD genes and looked for structural rearrangements within those genes that would explain known pathomechanisms of the disease (51 genes, Supplementary Materials 1). In the final step, we looked for genes that are ubiquitously expressed in pituitary. From the Protein Atlas we retrieved a list of 353 genes that showed high expressivity in pituitary (Supplementary Materials 2). Those genes were also thoroughly checked for structural abnormalities. The first attempt resulted in identification of copy gains of *HESX1* (one patient), *LHX4* (one patient), and *OTX2* (three patients). The evaluation of pituitary expressed genes resulted in identification of gains of *SIX3* transcription factor gene in two patients. In two other patients, we identified a heterozygous deletion of a region encompassing *ASH1L* gene and *POU5F1P4* pseudogene. Clinical characteristics as well as genomic coordinates are included in Table 1 and Table 2, whereas microarray rearrangements are shown on Figure 1.

## 5. Discussion

The deleterious effect of genes abundantly and exclusively expressed in the pituitary (like *POU1F1* or *LHX3*) is predictable and as expected, clinically relevant. An excellent review summarizing molecular mechanisms governing pituitary development was published [5,6,18].

However, the list of such genes is short, and the strategy of finding causative factors among only tissue specific proteins seems to be insufficient. In contrast, the effect of a surplus copy of a perfectly good gene is usually not regarded as potential source of disturbances. Although, there are also multiple examples of devastating effect of protein aggregates (i.e., *SNCA* gene and Parkinson disease) indicating particular importance of efficacy of endogenous proteasomal apparatus within the cell [19]. More data are coming from embryology indicating morphogens that are acting in a concentration-dependent manner [20]. Eventually, a dosage sensitive genes with striking example of *PMP22* gene, in regard to gene haploinsufficiency or an extra copy can cause varying phenotypes (HNPP or CMT disease respectively) [21]. For hypopituitarism, a duplication of Xq26-27 and *SOX3* gene was shown to be causative and dosage sensitive [14]. Surprisingly in this case, the authors prove that both copy loss and gain of the *SOX3* gene result in similar clinical manifestation, including infundibular hypoplasia and hypopituitarism.

**Figure 2:**
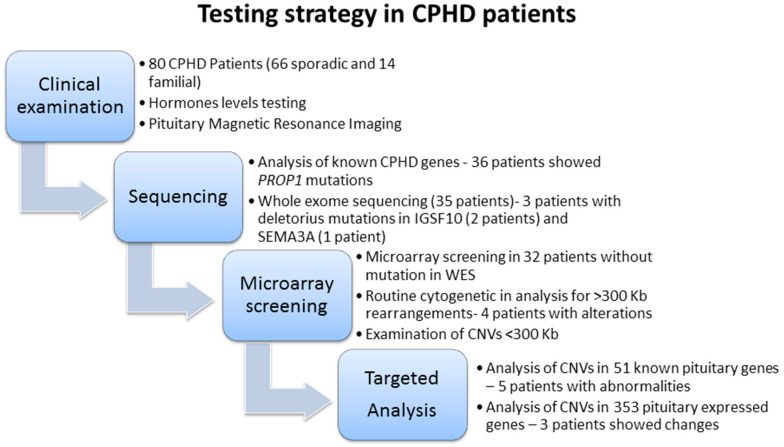
Testing strategy in CPHD patients.

Furthermore, functional studies on animal models evidencing that a delicate balance of signaling is crucial for pituitary cell fate specification or shaping. Expression disturbances of only one factor presenting loss-of-function or an opposite gain-of-function may trigger cascade of changes in expressivity of interacting molecules in specifically localized cells. Such developmental disturbances were evidenced for BMP4/SHH signaling [22], or more recently for *β*-catenin/WNT morphogenesis [23,24]. In our study, we refer to cases that have already been explored for known CPHD genes as well as throughout the exome. Despite this applied approach, we could not identify a causative pathogenic mutation in almost half of cases. Hence, we looked for genome-wide copy number variability with particular attention on pituitary expressed genes (strategy shown on Figure 2). For nine patients, we identified genomic gains. Involvement of the region encompassing the *LHX4* gene was found in one CPHD patient presenting PSIS. The *LHX4* gene encodes a protein, which contains the LIM domain, a unique cysteine-rich zinc-binding domain and is well characterized transcription factor involved in the control of development and differentiation of the pituitary gland [25,26]. The reported alterations indicated an autosomal dominant mode of inheritance and significant phenotypical variability among patients [27]. With regard to pituitary morphology, *LHX4* mutations were found in patients with hypoplasia, hyperplasia and PSIS [28]. Similar phenotypical variability is linked to another CPHD gene-*OTX2*. To the spectrum of pituitary phenotype, mutations in this gene brought severe ocular malformations as reported for selected cases [29,30]. In our study, none of the patients revealed any extra pituitary malformations. An interesting finding that came out from analysis of pituitary specific genes (Supplementary Files 2) is an involvement of copy number alteration encompassing sine oculis homeobox homolog 3 (SIX3). A *SIX3* gene includes a divergent DNA-binding homeodomain and an upstream SIX domain, which playing a role in DNA-binding specificity and in mediating of protein–protein interactions [31]. The deleterious effect of alterations is responsible for severe phenotype affecting brain development, namely holoprosencephaly and schizencephaly [32]. The gene itself is short, contains only two coding exons and is showing an abundant expression in developing and mature pituitary [33,34]. So far, the *SIX3* gene has not been linked to CPHD despite a strong evidences from experimental studies and resemblance to HESX1 phenotype [35,36]. In a study conducted by Gaston-Massuet [37], as well as in our previous report [16], we did not find any mutations in *SIX3* among CPHD patients and an evident haploinsufficiency. Authors stated, however, that due to limitation of sequencing method, the presence of bigger genomic rearrangements encompassing *SIX3* or its regulatory elements cannot be ruled out. In the present study, we identified duplication of 2p21 encompassing entire *SIX3* in two patients with pituitary hypoplasia. Although the impact of an extra copy of *SIX3* on tissue expression pattern or interacting targets (i.e., *HESX1*) need further elucidation. Vetro et al. have already reported a bigger genomic duplication in 2p25.3 [11]. This region was linked to syndromic hypopituitarism and classified as a pathogenic in a patient presenting IGHD and congenital defects including micropenis and bilateral cryptorchidism. The patient presented hypogonadotropic hypogonadism and MRI of pituitary revealed anterior lobe hypoplasia, posterior lobe ectopy, and thin stalk. The condition was caused by a complex rearrangement encompassing 14,7 Mb duplication of 2p25.3-p24.3 and 4 Mb deletion of 4q35.1-q35.2.

In two of our patients, the detected abnormality in 2p25.3 was significantly shorter (Patient 5: 0,53 Mb and Patient 6: 0,47 Mb), encompassing only eight genes (*TSSC1, TRAPPC12, ADI1, RNASEH1, LOC100506054, RPS7, COLEC11, ALLC*). Regarding an overlapping phenotype of our patients and those reported by Vetro et al. [11], we could further delineate and narrow down the loci linked to pituitary dysfunction bearing only eight genes. In contrast to genomic gains, losses were found for only two regions: 1q22 (1 patient) and 18q12.3 (2 patients). On chromosome 1, we identified a small 50 Kb heterozygotic deletion affecting *ASH1L* gene and a pseudogene *POU5F1P4*. The phenotype linked to histone methyltransferase *ASH1L* included a broad range developmental disturbances and intellectual disability (including autism spectrum), global developmental delay, and presenting autosomal dominant transmission.

Solute carrier family 14 member 2 gene (SLC14A2) belongs to the urea transporter family. So far, no abnormal phenotypes have been linked to the gene. In conclusion, we identified CNV in 12 out of 35 unrelated CPHD patients. We showed that CNVs abnormalities might be found not only in patients with complex severe phenotypes, but also in CPHD patients. The microarray approach could be regarded for CPHD patients, if sequencing failed in identification of causative mutations. Although these findings need to be confirmed by analysis of a bigger cohort of patients. The dosage-sensitivity of presented CNVs and mechanisms of the disease required thorough functional examination that would support causativeness and clinical relevancy of presented abnormalities.

## Figures and Tables

**Table 1:** Genomic regions identified in CPHD patients.

Patient	Type	Cytoband	Size (Kbp)	Gene Number	Gene Name	HGVS
1	Loss	1q22	54.284	2	ASH1L, POU5F1P4	arr[hg19] 1q22(155,394,484-155,448,768)x1
2	Gain	1q25.2	95.535	4	QSOX1, FLJ23867, LHX4, LOC100527964	arr[hg19] 1q25.2(180,143,701-180,239,236)x3
3	Gain	2p21	23.746	1	SIX3	arr[hg19] 2p21(45,151,117-45,174,863)x3
4	Gain	2p21	27.832	1	SIX3	arr[hg19] 2p21(45,151,117-45,178,949)x3
5	Gain	2p25.3	536.643	8	TSSC1, TRAPPC12, ADI1, RNASEH1, LOC100506054, RPS7, COLEC11, ALLC	arr[hg19] 2p25.3(3,232,368-3,769,011)x3
6	Gain	2p25.3	475.292	8	TSSC1, TRAPPC12, ADI1, RNASEH1, LOC100506054, RPS7, COLEC11, ALLC	arr[hg19] 2p25.3(3,339,014-3,814,306)x3
7	Gain	3p14.3	131.223	3	IL17RD, HESX1, APPL1	arr[hg19] 3p14.3(57,149,424-57,280,647)x3
8	Gain	14q22.3	46.766	2	OTX2	arr[hg19] 14q22.3(57251373-57298139)x3
9	Gain	14q22.3	47.781	2	OTX2	arr[hg19] 14q22.3(5763675-57311456)x3
10	Gain	14q22.3	24.896	2	OTX2	arr[hg19] 14q22.3(57263675-57288571)x3
11	Loss	18q12.3	373.468	1	SLC14A2	arr[hg19] 18q12.3(42,689,695-43,063,163)x1
12	Loss	18q12.3	390.92	1	SLC14A2	arr[hg19] 18q12.3(42,681,091-43,072,011)x1

**Table 2:** Clinical characteristics of studied CPHD patients with genes covered by detected rearrangements.

Case	Gene(s)	Gender	Age at Diagnosis	Pituitary Hormone Deficiency					MRI of Pituitary
				GH	Gn	TSH	PRL	ACTH	
1	ASH1L, POU5F1P4	M	13 y.o.	+	+	+	−	+	Pituitary hypoplasia
2	QSOX1, FLJ23867, LHX4, LOC100527964	F	9 y.o.	+	+	+	−	−	PSIS
3	SIX3	M	12 y.o	+	+	+	−	+	Pituitary hypoplasia
4	SIX3	M	15 y.o	+	+	+	+	+	Pituitary hypoplasia
5	TSSC1, TRAPPC12, ADI1, RNASEH1, LOC100506054, RPS7, COLEC11, ALLC	M	12 y.o	+	+	+	−	−	Pituitary hypoplasia
6	TSSC1, TRAPPC12, ADI1, RNASEH1, LOC100506054, RPS7, COLEC11, ALLC	F	24 y.o.	+	+	+	−	−	PSIS
7	IL17RD, HESX1, APPL1	M	12 y.o.	+	+	+	−	+	Pituitary hypoplasia
8	OTX2	F	10 y.o.	+	+	+	−	+	PSIS
9	OTX2	F	6 y.o.	+	+	−	−	−	Pituitary hypoplasia
10	OTX2	M	15 y.o.	+	+	+	−	−	Pituitary hypoplasia
11	SLC14A2	F	8 y.o.	+	+	+	−	−	Pituitary hypoplasia
12	SLC14A2	F	8 y.o.	+	+	+	−	−	Pituitary hypoplasia

## Supplementary Materials

The following are available online at https://www.mdpi.com/1422-0067/21/16/5757/s1.
